# Supplementary material for: Engineering of Bacillus Promoters Based on Interacting Motifs between UP Elements and RNA Polymerase (RNAP) α-Subunit
Source: Int J Mol Sci. 2022 Nov 3;23(21):13480. doi: 10.3390/ijms232113480 (PMC9655642; doi:10.3390/ijms232113480)
Supplement: Supplementary file 1 [file ijms-23-13480-s001.zip › Supplementary Table S1.pdf]

Supplementary Table S1. Primers used in this study

Primers	Sequence (5′-3′)
GFP-F	cccaagcttatgggtcgcggatccatggtagatctg
GFP-R	cgcgtcgactcacacgtggtggtggtggtggtgg
CTD-F	cgggatccgacgaagctcagcacgctga
CTD-R	gcgtcgactcaatcgtctttgcgaaggc
Primers for the amplification of native and predicted promoters	
HpaII-F	aaaaacgctttgcccaagcttttttgagtgatcttctcaaa
HpaII-R	ggatccgcgacccatacaaatgtgaggcattttcg
SpovG-F	aaaaacgctttgcccaagctttaagaaaagtgattctggga
SpovG-R	ggatccgcgacccatagtagttcaccaccttttcc
YdzA-F	aaaaacgctttgcccaagcttcgttctgttacagatggagg
YdzA-R	ggatccgcgacccataaatcgccctccttgtggac
P43-F	aaaaacgctttgcccaagcttagcattattgagtggatgat
P43-R	ggatccgcgacccatattcctctcttacctataat
TrxA-F	aaaaacgctttgcccaagcttaaaaagagcatatcccattc
TrxA-R	ggatccgcgacccattattgaattcctccaatgtg
GlvA-F	aaaaacgctttgcccaagcttagccctccggccaacccgta
GlvA-R	ggatccgcgacccataagccccttataagcgttt
Lan-F	aaaaacgctttgcccaagcttcatctcaattatacaaagaa
Lan-R	ggatccgcgacccattttccttcacctcttaatta
DS-F	aaaaacgctttgcccaagcttcgtttttcgccgaaatgca
DS-R	ggatccgcgacccatacaaacctcctttgaaattt
09-F	aaaaacgctttgcccaagcttgatcgtcacaatgcgccatc
09-R	ggatccgcgacccatggatcccactttatggacgc
Acpp-F	aaaaacgctttgcccaagcttggatcacgggccggccgcgg
Acpp-R	ggatccgcgacccattgtccaccatccgaactttt
R2-F	aaaaacgctttgcccaagctttgggctatagccaagcggta
R2-R	ggatccgcgacccatgttttatcttacctctgccc
Primers for construction of synthetic promoters with different flanking sequence lengths	
UP0-F	cccaagcttaagaaataatccaaaatagcccaaaaatagcgtgaacgaatgggaga
UP1-F	cccaagcttcattttgcacaagaaataatccaaaatagcccaaaaatagcgtgaacgaatgggaga
UP2-F	cccaagctttgaaatagggcattttgcacaagaaataatccaaaatagcccaaaaatagcgtgaacgaatgggaga
UP3-F	cccaagcttacaataattttgaaatagggcattttgcacaagaaataatccaaaatagcccaaaaatagcgtgaacgaatgggag a
UP4-F	cccaagcttacttattggacacaataattttgaaatagggcattttgcacaagaaataatccaaaatagcccaaaaatagcgtgaac gaatgggaga
UP5-F	cccaagcttcgaatatttaacttattggacacaataattttgaaatagggcattttgcacaagaaataatccaaaatagcccaaaaat agcgtgaacgaatgggaga
Primers for construction of synthetic promoters with repeated specific subsite in UP element	
UP5-1P-F	cccaagcttcgaatatttaacttattggacacaataattttgaaatagggcattttgcacaagaaataatccaaaatagccaaaata gc
UP5-2P-F	cccaagcttcgaatatttaacttattggacacaataattttgaaatagggcattttgcacaagaaataatccaaaatagccaaaata gccaaaatagc
UP5-3P-F	cccaagcttcgaatatttaacttattggacacaataattttgaaatagggcattttgcacaagaaataatccaaaatagccaaaata gccaaaatagccaaaatagc
UP5-4P-F	cccaagcttcgaatatttaacttattggacacaataattttgaaatagggcattttgcacaagaaataatccaaaatagccaaaata gccaaaatagccaaaatagccaaaatagc
UP5-5P-F	cccaagcttcgaatatttaacttattggacacaataattttgaaatagggcattttgcacaagaaataatccaaaatagccaaaata gccaaaatagccaaaatagccaaaatagccaaaatagc
UP5-1D-F	cccaagcttcgaatatttaacttattggacacaataattttgaaatagggcattttgggcattttg
UP5-2D-F	cccaagcttcgaatatttaacttattggacacaataattttgaaatagggcattttgggcattttgggcattttg
UP5-3D-F	cccaagcttcgaatatttaacttattggacacaataattttgaaatagggcattttgggcattttgggcattttgggcattttg

UP5-4D-F	cccaagcttcgaatatttaacttattggacacaataattttgaaatagggcattttgggcattttgggcattttgggcattttgggcatttttg
UP5-5D-F	cccaagcttcgaatatttaacttattggacacaataattttgaaatagggcattttgggcattttgggcattttgggcattttgggcatttttgggcattttg
Primers for construction of synthetic promoters with repeated specific subsite in UP element	
UP5-1P-F	cccaagcttcaaaatagctagcgtgaacgaatgggaga
UP5-2P-F	cccaagcttcaaaatagccaaaatagctagcgtgaacgaatgggaga
UP5-3P-F	cccaagcttcaaaatagccaaaatagccaaaatagctagcgtgaacgaatgggaga
UP5-4P-F	cccaagcttcaaaatagccaaaatagccaaaatagccaaaatagctagcgtgaacgaatgggaga
UP5-5P-F	cccaagcttcaaaatagccaaaatagccaaaatagccaaaatagccaaaatagctagcgtgaacgaatgggaga
Primers for construction of synthetic promoters with UP5-2P upstream of different parent promoters	
UPHpaII-F	cccaagcttcccaagcttcgaatatttaacttattggacacaataattttgaaatagggcattttgcacaagaaataatccaaaatagccaaaatagccaaaatagcccaaaaatagcgtgaacgaatgggagatttatgaatataaagtatag
UPSpovG-F	cccaagcttcccaagcttcgaatatttaacttattggacacaataattttgaaatagggcattttgcacaagaaataatccaaaatagccaaaatagccaaaatagcccaaaaatagcgtgaacgaatgggagatttaaaaacgagcaggattt
UPYdzA-F	cccaagcttcccaagcttcgaatatttaacttattggacacaataattttgaaatagggcattttgcacaagaaataatccaaaatagccaaaatagccaaaatagcccaaaaatagcgtgaacgaatgggagattgattttgtacacattatc
UPP43-F	cccaagcttcccaagcttcgaatatttaacttattggacacaataattttgaaatagggcattttgcacaagaaataatccaaaatagccaaaatagccaaaatagcccaaaaatagcgtgaacgaatgggagattgccaaagctgtaatggct
UPGlvA-F	cccaagcttcccaagcttcgaatatttaacttattggacacaataattttgaaatagggcattttgcacaagaaataatccaaaatagccaaaatagccaaaatagcccaaaaatagcgtgaacgaatgggagattgaaacagtagggtaaatt
UPDS-F	cccaagcttcccaagcttcgaatatttaacttattggacacaataattttgaaatagggcattttgcacaagaaataatccaaaatagccaaaatagccaaaatagcccaaaaatagcgtgaacgaatgggagattccctgttatagaatggtg
UP09-F	cccaagcttcccaagcttcgaatatttaacttattggacacaataattttgaaatagggcattttgcacaagaaataatccaaaatagccaaaatagccaaaatagcccaaaaatagcgtgaacgaatgggagattgacgacaagaacgtcctg
UPAcpp-F	cccaagcttcccaagcttcgaatatttaacttattggacacaataattttgaaatagggcattttgcacaagaaataatccaaaatagccaaaatagccaaaatagcccaaaaatagcgtgaacgaatgggagattgcaattagaaggcaatga
UPR2-F	cccaagcttcccaagcttcgaatatttaacttattggacacaataattttgaaatagggcattttgcacaagaaataatccaaaatagccaaaatagccaaaatagcccaaaaatagcgtgaacgaatgggagattccatctacaccctgcctg
Primers for the amplification of synthetic promoters	
fusionHpaII-F	AAAAACGCTTTGCCCAAGCTTGGGCAGGTTTTTTTGTTTCT
fusionSpovG-F	AAAAACGCTTTGCCCAAGCTTATTCTGGGAGAGCCGGGATC
fusionYdzA-F	AAAAACGCTTTGCCCAAGCTTTTTCTGCCTAATTCCCTCAT
fusionP43-F	AAAAACGCTTTGCCCAAGCTTTTTTTGCCGTGATTTCGTGT
fusionGlvA-F	AAAAACGCTTTGCCCAAGCTTAATTGTCTCTATTCTACACG
Primers for the amplification of synthetic promoters	
fusionHpaII-F	AAAAACGCTTTGCCCAAGCTTGGGCAGGTTTTTTTGTTTCT
fusionSpovG-F	AAAAACGCTTTGCCCAAGCTTATTCTGGGAGAGCCGGGATC
fusionYdzA-F	AAAAACGCTTTGCCCAAGCTTTTTCTGCCTAATTCCCTCAT
fusionP43-F	AAAAACGCTTTGCCCAAGCTTTTTTTGCCGTGATTTCGTGT
fusionGlvA-F	AAAAACGCTTTGCCCAAGCTTAATTGTCTCTATTCTACACG
fusionLanA-F	AAAAACGCTTTGCCCAAGCTTAGTTTATGTATAGATATTTT
fusionDS-F	AAAAACGCTTTGCCCAAGCTTCGTTTTTTCGCCGAAATGCA
fusion09-F	AAAAACGCTTTGCCCAAGCTTGATCGTCACAATGCGCCATC

fusionAcpp-F	AAAAACGCTTTGCCCAAGCTTTTTTCGTCTATAAAGATAAA
fusionR2-F	AAAAACGCTTTGCCCAAGCTTTTACATAAGGTGTCTTTTTT
Primers labeled for EMSA or FP	
UPSpovG-F	TAAGAAAAGTGATTCTGGGAGAGCC
UPSpovG-R (5'-Biotin)	AGTAGTTCACCACCTTTTCCCTATA
UPTrxA-F	AAAAAGAGCATATCCCATTCAACCA
UPTrxA-R (5'-Biotin/FAM)	TATTGAATTCCTCCAATGTGAAATG
UPLanA-F	CATCTCAATTATACAAAGAAGGAAA
UPLanA-R (5'-Biotin)	TTTCCTTCACCTCTTAATTAATTTT
UP09-F	GATCGTCACAATGCGCCATCAAACC
UP09-R (5'-Biotin)	GGATCCCACTTTATGGACGCCGCAG
