# Supplementary figures and images for: Engineering of Bacillus Promoters Based on Interacting Motifs between UP Elements and RNA Polymerase (RNAP) α-Subunit
Source: Int J Mol Sci. 2022 Nov 3;23(21):13480. doi: 10.3390/ijms232113480 (PMC9655642; doi:10.3390/ijms232113480)

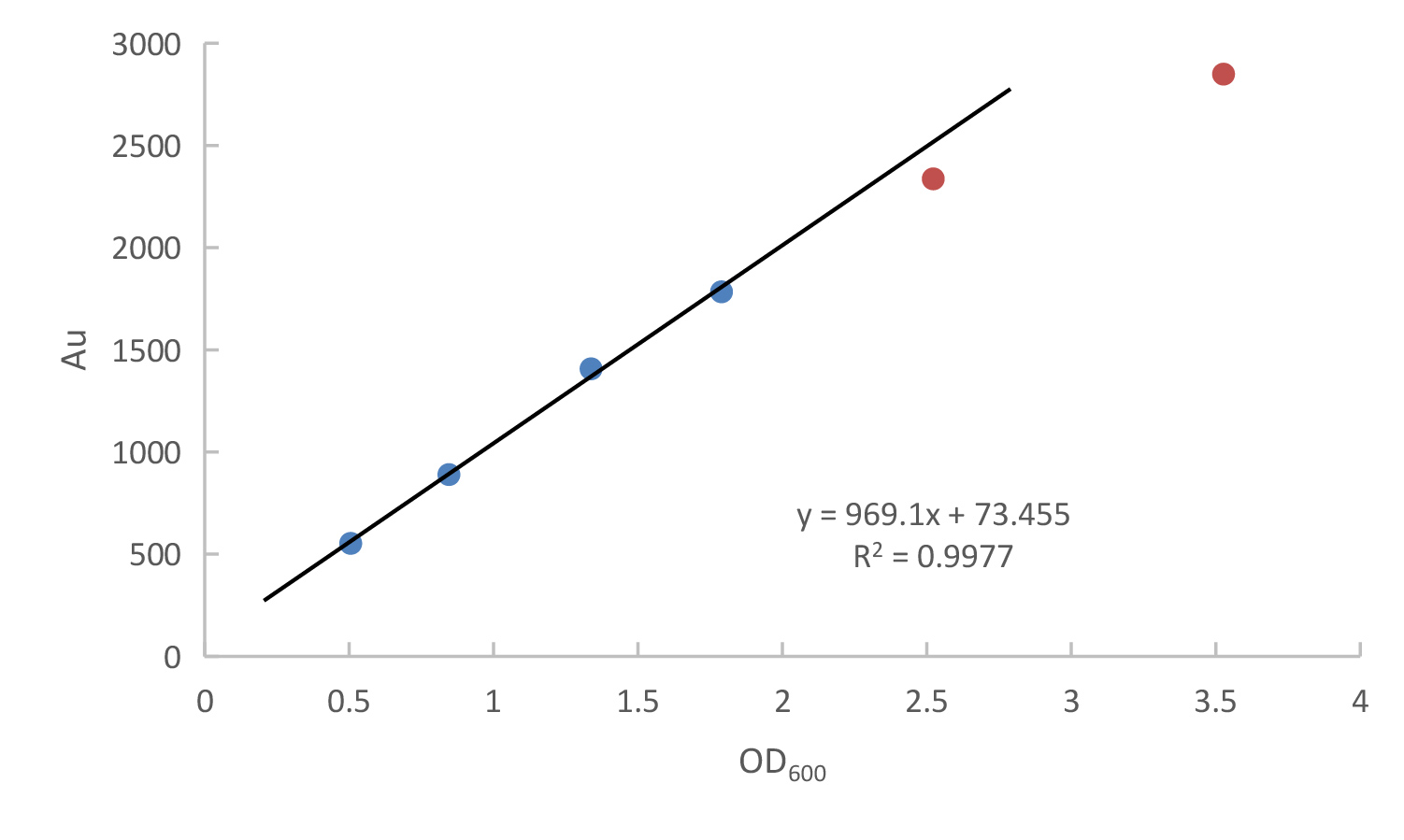

Supplement: Supplementary file 1 [file ijms-23-13480-s001.zip › Supplementary Figure S1.jpg]
